# Editorial: Sports nutrition and sustainability: Steps towards a healthier planet

**DOI:** 10.3389/fspor.2023.1146970

**Published:** 2023-02-24

**Authors:** Amira M. Galal Darwish, Hesham Ali El Enshasy, Mohamed A. E. Gomaa, Marina Mefleh

**Affiliations:** ^1^ Food Industry Technology Program, Faculty of Industrial and Energy Technology, Borg Al Arab Technological University (BATU), Alexandria, Egypt; ^2^ Food Technology Department, Arid Lands Cultivation Research Institute (ALCRI), City of Scientific Research and Technological Applications (SRTA-City), Alexandria, Egypt; ^3^ School of Chemical and Energy Engineering, Faculty of Engineering, Universiti Teknologi Malaysia (UTM), Johor Bahru, Johor, Malaysia; ^4^ Genetic Engineering & Biotechnology Research Institute (GEBRI), City of Scientific Research and Technology Application (SRTA-City), New Burg Al Arab, Alexandria, Egypt; ^5^ Institute of Bioproduct Development (IBD), Universiti Teknologi Malaysia (UTM), Johor Bahru, Johor, Malaysia; ^6^ Department of Food Science, Faculty of Agriculture, Alexandria University, Saba Basha, Egypt; ^7^ Department of Soil, Plant and Food Science (Di.S.S.P.A.), University of Bari Aldo Moro, Bari, Italy

**Keywords:** editorial, good health and wellbeing, nutrition, sustainability, protein, diet

**Editorial on the Research Topic** Sports nutrition and sustainability: Steps towards a healthier planet

Since 2015, the Paris Agreement required all parties to put forward their best efforts through nationally determined contributions (NDCs), embody efforts by each country to reduce national emissions and adapt to the impacts of climate change (1). Global greenhouse gas emissions caused by human activities have substantially increased during the last decades, and food systems activities are responsible for about one-third of these emissions especially animal agriculture which is responsible for 14.5% of global greenhouse gas emissions according to the UN and FAO. Although athletes have higher protein demands than the general population, negative environmental outcomes of sports participation have received little attention paired with a lack of knowledge and awareness. Coinciding with the latest 27th United Nations Climate Change Conference (COP27), held in Sharm El-Sheikh, Egypt (2022), these neglected fields should be highlighted (2).

With just 8 years to go, ambitious global efforts are underway to deliver the Sustainable Development Goals SDGs by 2030 through raising awareness and mobilizing more governments, civil society, and businesses sectors including the food sector that should align with the SDGs. The alternative protein field needs more scientists driving academic research for better alternatives to conventional meat, egg, and dairy products. There are many alternatives such as plant-, insect-, fungi-, algae-, fermented-based proteins, and cultivated meat. Furthermore, fermented foods also contribute to environmental sustainability by making use of available local production with minimal additional agricultural input and little energy compared with food processing methods, especially in low-income regions. Currently, athletes are also interested in fermented products as a significant source of nutrients, including proteins; essential fatty acids, soluble fiber, minerals, vitamins, and essential amino acids, in addition to their prebiotic content, and the presence of specific lactic acid bacterial strains (LAB) that combat gut imbalances, increase protein synthesis and mineral bioavailability providing a beneficial impact in workout nutrition (3, 4).

Thus, the Special Issue focus on specific healthy food ingredients, supplements, or habitual diets with new perspectives, sources, or techniques involving statistical, cell culture, animals, or human models, especially research topics dealing with various interactions including; maximizing protein intake, synthesis and availability, consumer acceptance, safety consideration studies and greenhouse gas emissions throughout the supply chain. The four quality papers; three research articles and one review, published in this Special Issue exhibited its aims with varied perspectives.

The article “*Protein and sport: Alternative sources and strategies for bioactive and sustainable sports nutrition*”, summarized the latest research on greener and functional supplementation of proteins in sport nutrition such as, alternative protein, peptides, amino acids sources and protein hydrolysis as a useful technology to revalue by-products (5, 6). The demand for alternative proteins has been growing considerably and it is estimated that it will continue to do so in the next 20 years. In this context, sports nutrition will be an interesting target market and those new products that combine protein quality and bioactivity, including ergogenic capacity, and are friendly to the environment will be better accepted by athletes. Although alternative proteins present a great potential as natural source of biologically active compounds, the scientific evidence related to the biological properties of new sources of protein is almost limited to *in vitro* assays and it will be necessary more studies in animal models and human trials to evaluate their efficacy, safety, and their impact in human health before its commercialization.

The research article “*Exercise-induced browning of white adipose tissue and improving skeletal muscle insulin sensitivity in obese/non-obese growing mice: Do not neglect exosomal miR-27a*”, explored the possible cause of exercise affecting white adipose tissue (WAT) browning and reversing skeletal muscle insulin resistance in obese/non-obese immature bodies. They indicated that exosomal miR-27a might be a crucial node for the process of exercise-induced browning of WAT and improving skeletal muscle insulin sensitivity as the root cause of irregular or excessive fat accumulation distinguished in children with obesity, is due to imbalance between energy intake and expenditure (7). For simulating the process of childhood obesity, juvenile mice were fed with a basal diet or high-fat diet (HFD) and took 1 or 2 h swimming exercise simultaneously for 10 weeks. The obese animal model was induced by the HFD. The exercise of hindered HFD-induced body fat was developed in growing mice. Exercise modified glucolipid metabolism parameters differently in the obese/non-obese groups, and the changes of the 2 h exercise mice were not consistent with the 1 h exercise mice. The level of serum exosomal miR-27a in the non-exercise obese group was increased obviously, which was reduced in the exercise obese groups. Results from bioinformatics analysis and dual-luciferase reporter assay showed that miR-27a targeted PPAR-*γ*. Exercise stimulated WAT browning; however, the response of obese WAT lagged behind normal WAT. In the HFD-fed mice, 2 h exercise activated the IRS-1/Akt/GLUT-4 signaling pathway in the skeletal muscles. The findings confirmed that exercise-induced beneficial effects are associated with exercise duration, and the response of obese and non-obese bodies is different (8).

The American dietetic association (ADA) (9) recommended a protein intake of 1.2–2.0 g per kilogram (kg) of body mass for adult athletes, from good quality protein sources divided into moderate portions throughout the day. It is a common belief among athletes that sources of animal protein are needed for muscle recovery (10). The adequacy of low-carbon diets that meet the protein requirements of adolescent athletes through a cross-sectional study conducted with 91 adolescent athletes from Rio de Janeiro was invastigated by the article “*Effectiveness of current protein recommendations in adolescent athletes on a low-carbon diet*”. The candidates underwent anthropometric and food consumption assessments. To estimate the environmental impact of anthropogenic activities, the sustainability indicators carbon footprint (CF) and water footprint (WF) were used. The CF of the athlete's diet was compared with the benchmark of 1,571 g CO2eq/cap/d estimated by the World Wildlife Fund (WWF). Protein recommendations according to the American Dietetic Association (ADA) for athletes and protein food groups according to the low-carbon EAT-Lancet reference diet were used as references. The results indicated that a balanced diet can be designed for athletes with a plant-to-animal protein ratio of 3 ×  1 with a lower calorie intake, which can compromise sports performance. Future research should evaluate athletes’ diets in terms of sustainability and sports performance ensuring less negative environmental impacts of food production and consumption.

Whey is a rich source of high-quality proteins; especially branched amino acids (leucine, isoleucine, and valine) that enhance muscle protein synthesis (11). On the other hand, beetroot wastes proved their functional and nutraceutical potential such as; extended the shelf life (12) and enhanced nutritional quality of juice (13). *Valorization of whey proteins and beetroot peels to develop a functional beverage high in proteins and antioxidants*, exhibited developed functional beverage based on mixing whey protein isolate with beetroot peel water extract flavored with strawberries puree. Whey protein isolate enriched the juices with stable protein content during the storage (4.65%–4.69%). Beverages with 2.5% peel extract showed preferable sensory attributes and increased phenolic and flavonoids by 44% and 31% which elevated the scavenging activity of the juice. Results highlighted the role of these supplements in providing the body with high-quality protein and considerable amounts of antioxidants. The developed beverage could be recommended as a suitable and safe source of protein and antioxidants for individuals, particularly athletes, that could effectively benefit their health.

We are seeking that this Special Issue will further raise the awareness to keep our plant healthier by shedding the light on ecofriendly foods such as; functional and animal-based protein alternatives targeting sustainability and healthy life style.
